# H-NS Can Facilitate Specific DNA-binding by RNA Polymerase in AT-rich Gene Regulatory Regions

**DOI:** 10.1371/journal.pgen.1003589

**Published:** 2013-06-20

**Authors:** Shivani S. Singh, David C. Grainger

**Affiliations:** Institute for Microbiology and Infection, School of Biosciences, University of Birmingham, Edgbaston, Birmingham, United Kingdom; Institute of Molecular and Cell Biology (IMCB), A*STAR, Singapore

## Abstract

Extremely AT-rich DNA sequences present a challenging template for specific recognition by RNA polymerase. In bacteria, this is because the promoter −10 hexamer, the major DNA element recognised by RNA polymerase, is itself AT-rich. We show that Histone-like Nucleoid Structuring (H-NS) protein can facilitate correct recognition of a promoter by RNA polymerase in AT-rich gene regulatory regions. Thus, at the *Escherichia coli ehxCABD* operon, RNA polymerase is unable to distinguish between the promoter −10 element and similar overlapping sequences. This problem is resolved in native nucleoprotein because the overlapping sequences are masked by H-NS. Our work provides mechanistic insight into nucleoprotein structure and its effect on protein-DNA interactions in prokaryotic cells.

## Author Summary

The information required to build and maintain a cell is written into an organism's DNA in the form of genes. When individual genes are “read,” the DNA code is transcribed into an mRNA molecule by RNA polymerase. Hence, the DNA sequence adjacent to the start of a gene must contain a signal to recruit RNA polymerase. In certain instances this signal is difficult to differentiate from the background DNA sequence. For example, many bacterial chromosomes contain discrete sections of DNA with a high percentage of A and T nucleotides. Because RNA polymerase recognises an AT-rich signal sequence, these chromosomal regions can be ambiguous. In this paper we address the long-standing question of how RNA polymerase specifically recognises such DNA target sites. We show that a crucial factor is local nucleoprotein organisation. Hence, the manner in which DNA is folded, in conjunction with primary DNA sequence, facilitates specific RNA polymerase interactions with DNA.

## Introduction

Transcription is initiated by binding of RNA polymerase to specific DNA sequences known as promoters [1]. Following promoter recognition the resulting complex undergoes a process of isomerisation. Hence, ∼14 base pairs (bp) of DNA, close to the transcription start site, are unwound [2]. RNA polymerase then engages in abortive cycles of initiation before escaping the promoter to form an elongation complex [3]. It has long been known that promoter unwinding is facilitated by the weak base stacking interactions associated with AT-rich DNA. Thus, the eukaryotic TATA box (5′-TATAAA-3′) is unwound during transcription initiation [4]. Similarly, the prokaryotic −10 hexamer (5′-TATAAT-3′), recognised by Domain 2 of the RNA polymerase σ^70^ subunit, participates in DNA opening [5]. Because DNA elements recognised by RNA polymerase are AT-rich, chromosomal regions, where DNA AT-content is unusually high, prove particularly challenging templates for recognition. For example, the horizontally acquired sections of some bacterial chromosomes have an elevated AT-content. As a result, RNA polymerase may bind cryptic promoters [6] or initiate transcription promiscuously [7].

In *Escherichia coli*, gene regulatory regions are targeted by chromosome folding proteins [8]. Hence, in addition to their architectural role, these proteins can influence RNA polymerase-DNA interactions [9]. The Histone-like Nucleoid Structuring (H-NS) protein recognises AT-rich DNA and is associated with horizontally acquired genes [10]–[13]. The prevailing view is that, when bound at such regions, H-NS silences transcription [14]. However, the precise mechanism remains elusive; models proposing exclusion of RNA polymerase from, and trapping of RNA polymerase at, H-NS bound regions have both been proposed [15]. Since these models are not mutually exclusive a third possibility is that a myriad of different configurations exist. Interestingly, two recent studies have reported close association between RNA polymerase and H-NS [16], [17]. In one case, H-NS stimulated rather than repressed gene expression [17].

In this work we describe an undocumented role for H-NS; facilitating the correct recognition of promoters by RNA polymerase. The *ehxCABD* operon from Shiga toxin-producing *E. coli* (STEC) has an unusually high AT-content. Consequently, the operon regulatory region contains multiple sequences that resemble −10 hexamers. We show that, despite the apparent ambiguity of this DNA template, RNA polymerase initiates transcription specifically from a single promoter *in vivo*. However, *in vitro*, RNA polymerase is unable to differentiate between this promoter and adjacent binding sites. We show that H-NS plays a critical role by blocking access of RNA polymerase to the adjacent binding sites. Thus, H-NS ensures correct positioning of RNA polymerase.

## Results

### Promoter activity locates to the upstream section of the*ehxCABD* gene regulatory region

**Figure 1:**
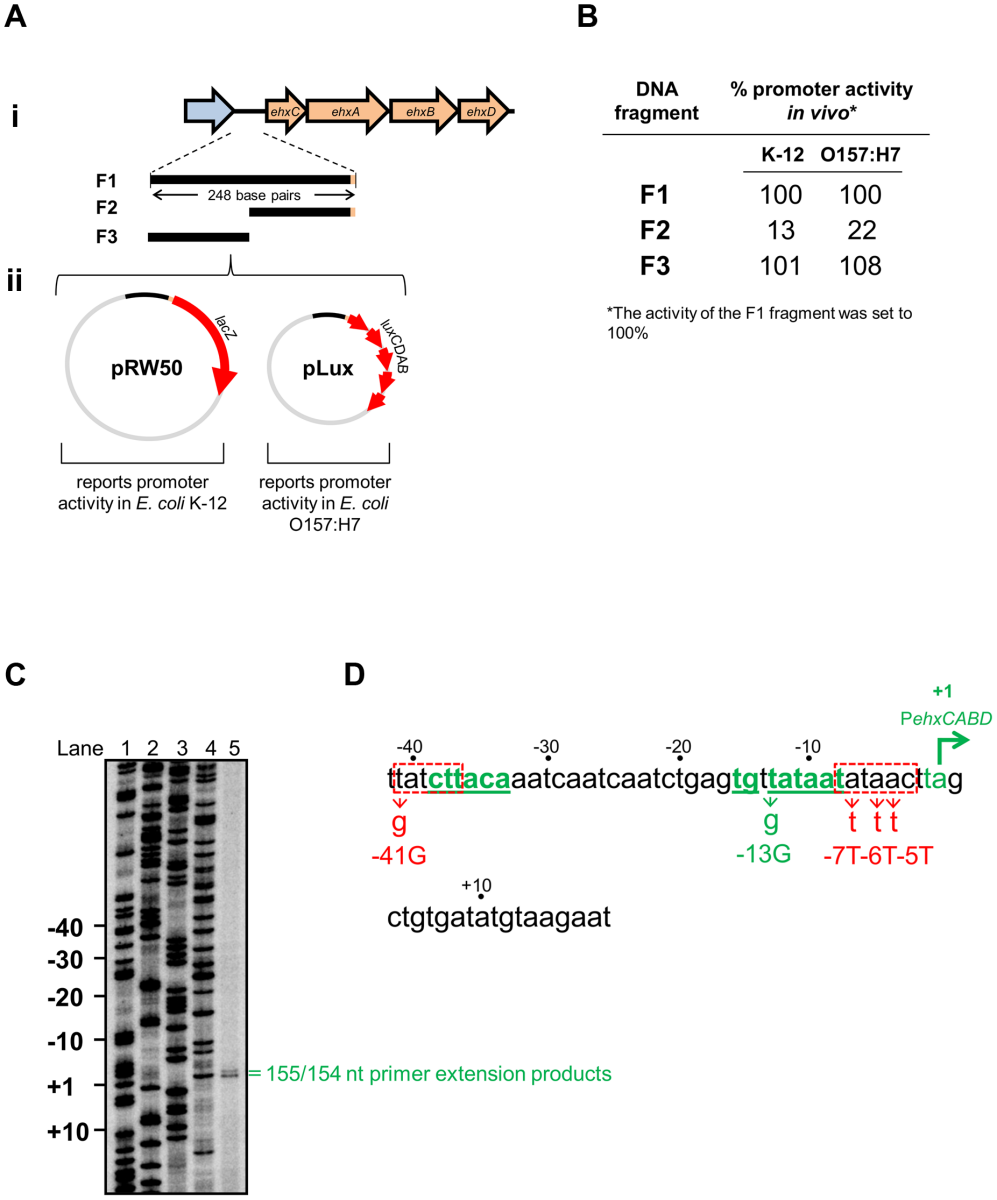
Identification of the*ehxCABD* promoter. **A. i) Schematic representation of the** ***ehxCABD*** **operon and gene regulatory region.** The figure shows genes (as block arrows) within the *ehxCABD* operon (orange). The adjacent open reading frame is coloured blue. The *ehxCABD* regulatory region fragments used in this study are shown as solid black lines labelled F1 through F3. The 248 bp F1 fragment contains regulatory DNA upstream of, and including, the *ehxC* start codon. The F3 and F2 fragments are equivalent to upstream and downstream parts of the F1 fragment respectively. **ii) Plasmid maps for pRW50 (containing a LacZ reporter) and pLux (containing a Luciferase reporter)** that were used to test the ability of the F1–F3 fragments to drive transcription. **B. Promoter activity of different** ***ehxCABD*** **regulatory DNA fragments.** The panel shows a summary of data from β-galactosidase and Luciferase assays using the different pRW50 and pLux constructs in *E. coli* strains JCB387 and O157:H7 respectively. The data are expressed as a percentage of the signal obtained for the F1 fragment. **C. Location of the** ***ehxCABD*** **transcription start site.** The gel shows products from an mRNA primer extension analysis of the F3 fragment (Lane 5). The gel was calibrated using arbitrary size standards (A, C, G and T in Lanes 1–4). **D. Location of the** ***ehxCABD*** **promoter.** The panel shows the base sequence of the non-template strand. The transcript start sites identified in panel A are highlighted in green with the most abundant start site labelled as “+1”. The proposed extended −10 and −35 hexamer elements of the *ehxCABD* promoter are also in green as well as being underlined. Two sequences that resemble a promoter −10 element are boxed by a dashed red line. The positions of mutations designed to disrupt the various RNA polymerase binding elements are also shown. The -41G mutation disrupts the highly conserved “T” that occurs in the first position of −10 elements. Concomitantly, the genuine P*ehxCABD* −35 hexamer remains intact. We made more conservative changes to disrupt the downstream −10 like sequence. This element is embedded within the region of P*ehxCABD* that participates in open complex formation. Thus, we made several A to T transversions to remove the problematic −10 like sequence whilst maintaining the AT-content of the DNA. We reasoned that this would be least disruptive to DNA opening during transcription initiation. However, we cannot rule out the possibility of small structural changes to P*ehxCABD*.

The *ehxCABD* operon is located on the pO157 plasmid and its derivatives. The operon encodes an enterohemolysin and proteins for its post-translational modification and export [18]. The 248 bp of regulatory DNA immediately upstream of the operon has an AT-content of 71%. H-NS has been implicated in regulating expression of the operon but a comprehensive molecular analysis is lacking [19]–[21]. As a first step we determined which section of the regulatory DNA contained promoter activity. Note that the *ehxCABD* regulatory DNA has an almost identical sequence in multiple *E. coli* serotypes and we arbitrarily used the *ehxCABD* regulatory sequence described by Rogers *et al.* [20]. We began by generating DNA fragments carrying discrete sections of the *ehxCABD* regulatory region (illustrated in Figure 1Ai). The fragments encompass 248 bp of DNA adjacent to the first gene in the operon (fragment F1), the downstream part of this region (fragment F2) or the upstream section of the locus (fragment F3). We assayed each fragment for promoter activity using two plasmid based systems (illustrated in Figure 1Aii). Hence, pRW50 and pLux encode the reporter proteins β-galactosidase and Luciferase respectively. Note that pRW50 was used to report promoter activity in *E. coli* K-12 whilst pLux was used with *E. coli* O157:H7 as a control for effects of STEC encoded transcriptional regulators. The raw activity data, for each DNA fragment, in each plasmid, is summarised in Figure 1B. Our results show that the F1 and F3 fragments stimulate transcription, to similar levels, in all of the assays. No detectable transcription was driven by the F2 fragment. Therefore, the *ehxCABD* promoter must be located in the upstream portion of the regulatory region common in both F1 and F3.

### RNA polymerase utilises a single promoter within the*ehxCABD* F3 fragment *in vivo*

Our next aim was to identify transcription start sites in the F3 fragment. To do this we conducted mRNA primer extension experiments. We used RNA extracted from *E. coli* JCB387 cells, carrying the F3 fragment cloned in plasmid pRW50. Our analysis yielded two extension products of 155 and 154 nucleotides (nt) in length (Figure 1C). The transcript start, corresponding to the more abundant 154 nt extension product, is labelled +1 in Figure 1D. A consensus extended promoter −10 element (5′-TGnTATAAT-3′) was found 8 bp upstream of the transcription start site. A four out of six match to a promoter −35 element (5′-TTGACA-3′) was observed further upstream. Throughout this work we refer to this promoter, highlighted green in Figure 1D, as P*ehxCABD*. The two primer extension products, differing in length by a single nt, both likely originate from this promoter. Importantly, we confirmed that P*ehxCABD* was the only promoter present in the F1 fragment. Thus, using RNA extracted from *E. coli* JCB387 cells carrying the F1 fragment cloned in plasmid pRW50, we observed only primer extension products corresponding to P*ehxCABD* (Figure S1).

### RNA polymerase binds multiple sites within the*ehxCABD* F3 fragment *in vitro*

**Figure 2:**
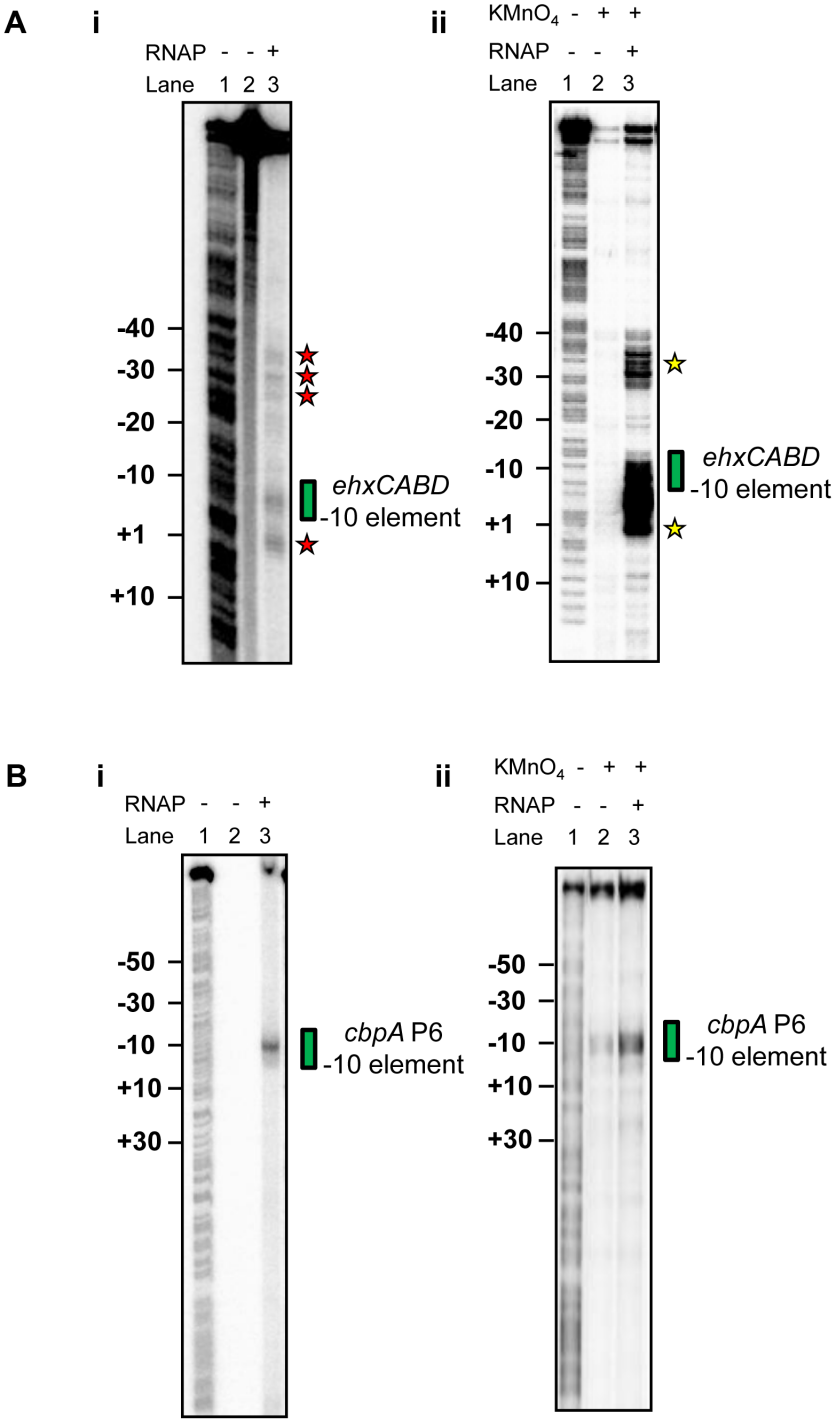
RNA polymerase binds multiple sites in the*ehxCABD* gene regulatory region. **A. i) Footprint of RNA polymerase (σ^70^ RC461-FeBABE) interactions with −10 elements in the** ***ehxCABD*** **regulatory region.** The gel shows DNA cleavage products resulting from incubation of the *ehxCABD* promoter F3 fragment with RNA polymerase containing σ^70^ RC461-FeBABE (640 nM). Note that σ^70^ RC461-FeBABE results in specific cleavage of promoter −10 elements. Cleavage of the P*ehxCABD* −10 element is indicated by a green box. Additional sites at which the DNA is cleaved are highlighted by red stars. The gel was calibrated with a G+A sequencing ladder (Lane 1). **ii) KMnO_4_ reactivity pattern of the** ***ehxCABD*** **promoter in the presence and absence of RNA polymerase.** The panel shows DNA cleavage products resulting from KMnO_4_ treatment of a complex formed between RNA polymerase (320 nM) and the *ehxCABD* F3 fragment. Thus, the sites of DNA cleavage correspond to DNA unwinding by RNA polymerase at −10 hexamers. The P*ehxCABD* −10 element is indicated by a green box. Additional sites at which the DNA is cleaved are highlighted by yellow stars. **B. i) Footprint of RNA polymerase (σ^70^ RC461-FeBABE) interactions with −10 elements in the** ***cbpA*** **regulatory region.** The image shows an identical set of reactions to those illustrated in Figure 4Ai except that a DNA fragment containing the *cbpA* P6 promoter was used. The *cbpA* P6 −10 hexamer is highlighted by a green box. **ii) KMnO_4_ reactivity pattern of the** ***cbpA*** **P6 promoter in the presence and absence of RNA polymerase.** The image shows an identical set of reactions to those illustrated in Figure 4Aii except that a DNA fragment containing the *cbpA* P6 promoter was used. The *cbpA* P6 −10 hexamer is highlighted by a green box.

Our primer extension analysis shows that, *in vivo*, RNA polymerase initiates *ehxCABD* transcription with precision (Figure 1). This is remarkable given the abundance of potential −10 hexamer sequences in this regulatory region (two such sequences are highlighted red in Figure 1D). To better understand how specificity is achieved we examined recognition of the naked F3 fragment by RNA polymerase. We utilised two *in vitro* DNA footprinting techniques. First, we exploited the properties of Fe^2+^ chelated Bromoacetamidobenzyl-EDTA (FeBABE). FeBABE is a DNA cleavage reagent that can be attached to specific cysteine side chains in proteins. Once attached, FeBABE cleaves nucleic acids within a 12 Å radius of the attachment site. Thus, FeBABE conjugated with the RC461 derivative of *E. coli* σ^70^ cleaves promoter −10 elements [22]. Figure 2Ai shows the pattern of FeBABE cleavage observed with the F3 fragment. As expected, the P*ehxCABD* −10 element was cleaved (highlighted by green box in Figure 2Ai). However, we also observed DNA cleavage at additional sites overlapping P*exhCABD* (highlighted by red stars in Figure 2Ai). In complementary experiments KMnO_4_ footprinting was used to detect DNA unwinding by RNA polymerase. We observed DNA melting at the P*ehxCABD* −10 element (highlighted by a green box in Figure 2Aii) and at additional sites (highlighted by yellow stars in Figure 2Aii). It did not escape our attention that the additional sites of FeBABE and KMnO_4_ reactivity align with each other and with sequences that resemble −10 hexamers highlighted in Figure 1D. Nevertheless, we were concerned that the additional FeBABE and KMnO_4_ reactivity signals might originate from RNA polymerase bound at P*ehxCABD*. To exclude this possibility we ran identical reactions with unrelated *cbpA* P6 promoter DNA. In these experiments no DNA cleavage products were observed other than those at the *cbpA* P6 −10 hexamer. We conclude that the naked P*ehxCABD* F3 fragment must contain multiple overlapping RNA polymerase binding sites.

### Co-binding of RNA polymerase and H-NS at the*ehxCABD* regulatory region

**Figure 3:**
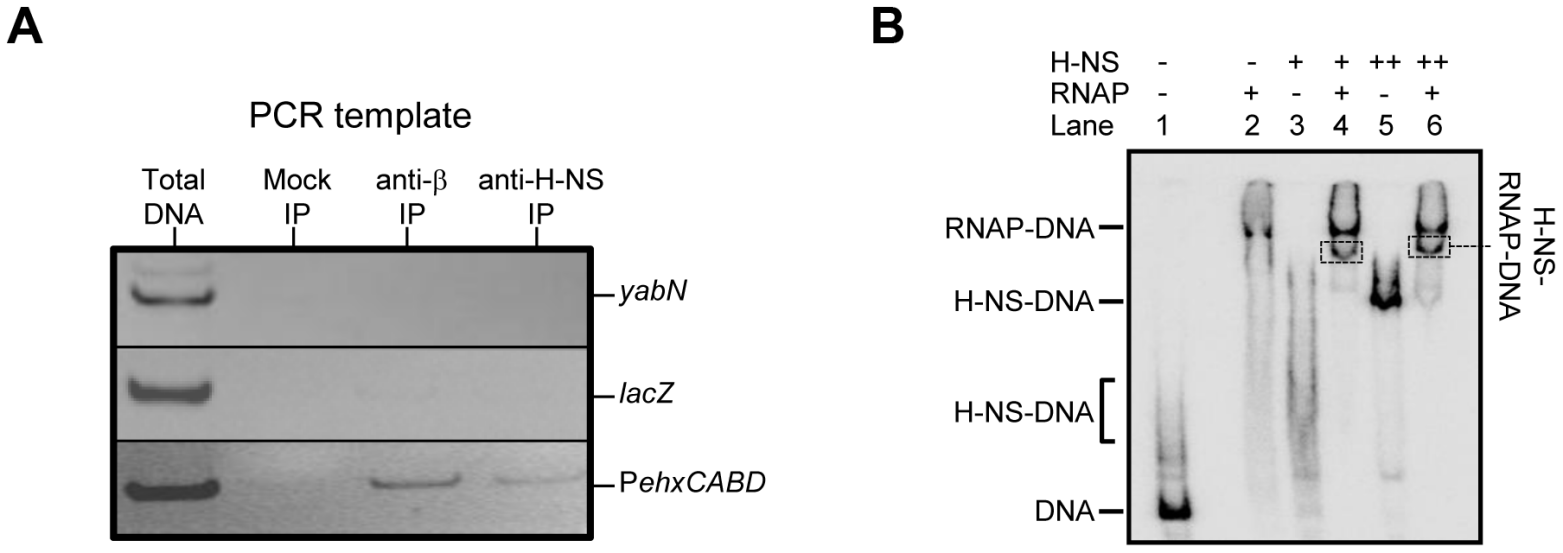
Co-association of RNA polymerase and H-NS with the*ehxCABD* gene regulatory region. **A. ChIP analysis of RNA polymerase and H-NS binding at the** ***ehxCABD*** **promoter.** The figure illustrates the result of a ChIP experiment designed to monitor the binding of H-NS and RNA polymerase to the *ehxCABD* F3 promoter fragment. The image shows a gel on which PCR products, generated with primers designed to detect P*ehxCABD*, *yabN* or *lacZ*, were analysed. The source of the DNA template (i.e. total cellular DNA or DNA from an immunoprecipitation) is shown above the gel image and the different PCR products are labelled to the right of the image. The mock immunoprecipitation contained no antibody. **B. EMSA analysis of H-NS and RNA polymerase binding at the** ***ehxCABD*** **promoter.** The results of an Electrophoretic Mobility Shift Assay (EMSA) are shown. The *ehxCABD* F3 DNA fragment (Lane 1) was incubated with 480 nM RNA polymerase (Lane 2), 2350 nM H-NS (Lane 3) or 4700 nM H-NS (Lane 5). The positions of the various H-NS-DNA and RNA polymerase-DNA complexes are indicated. Lanes 4 and 6 show complexes formed in the presence of 480 nM RNA polymerase and either 2350 nM or 4700 nM H-NS respectively. The bands highlighted by boxes were extracted and the presence of both H-NS and RNA polymerase proteins in the band was confirmed.

Factors present *in vivo* must influence RNA polymerase interactions with P*ehxCABD*. Such factors may explain why the additional RNA polymerase binding sites observed *in vitro* do not generate transcripts *in vivo*. Our attention turned to H-NS, which is known to recognise AT-rich regulatory regions and influences *ehxCABD* expression [19]–[21]. Thus, we used chromatin immunoprecipitation (ChIP) to measure binding of RNA polymerase and H-NS to P*ehxCABD in vivo*. Recall that, in ChIP experiments, a cell's nucleoprotein is cross-linked with formaldehyde, extracted, and then fragmented by sonication. Antibodies directed against the protein of interest are then used to select DNA fragments with which the protein is cross-linked. Finally, PCR is used to identify recovered DNA fragments. Figure 3A shows PCR analysis of DNA immunoprecipitated with anti-RNA polymerase (β subunit) or anti-H-NS. Control experiments, in which we analysed total cellular DNA, or DNA recovered from a mock immunoprecipitation, are also shown. The P*ehxCABD* DNA is detected in the total DNA sample, the anti-β, and anti-H-NS immunoprecipitates. Importantly, the P*ehxCABD* DNA was not detected in the mock immunoprecipitate. In a set of control PCR reactions we probed the *lacZ* and *yabN* loci. Note that these loci are not transcribed in the conditions used here and are not bound by H-NS. As expected, *lacZ* and *yabN* were not detected in the immunoprecipitates.

We next reconstituted co-association of RNA polymerase, H-NS and P*exhCABD in vitro*. Electrophoretic Mobility Shift Assays (EMSA) were used to probe the complexes formed. The result is shown in Figure 3B. The data show that RNA polymerase (lane 2) and H-NS (at two different concentrations, lanes 3 and 5) form distinguishable complexes with the DNA. When H-NS and RNA polymerase are added in unison an additional complex can be detected (boxed in lanes 4 and 6). To confirm that this additional complex contained both H-NS and RNA polymerase the band was extracted, submitted to tryptic digest, and the resulting peptides analysed by mass spectrometry. Both RNA polymerase and H-NS were present in the excised band.

### Correct positioning of RNA polymerase at P*ehxCABD* requires H-NS

**Figure 4:**
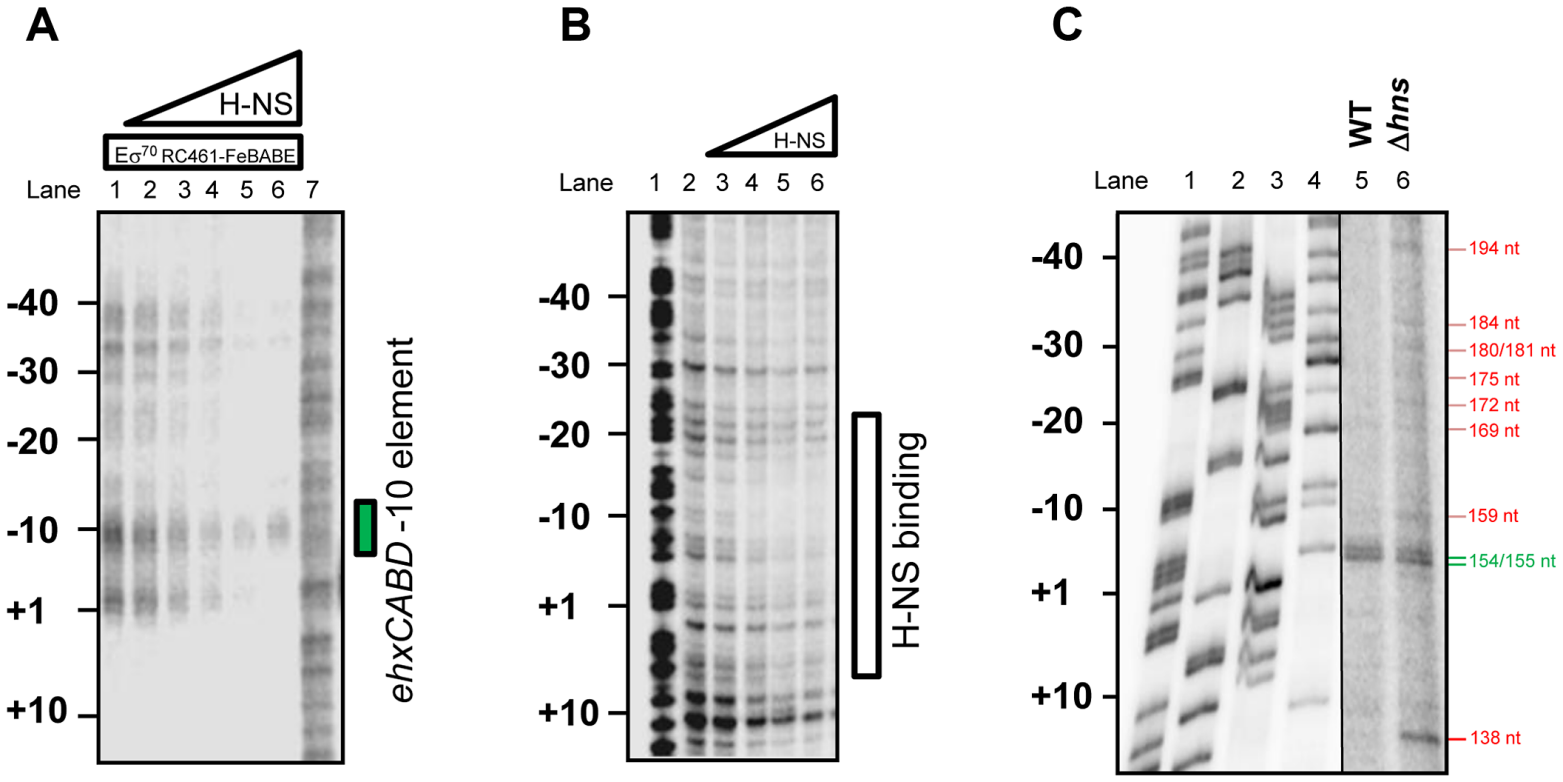
H-NS is required for correct positioning of RNA polymerase at P*ehxCABD*. **A. Footprint of RNA polymerase (σ^70^ RC461-FeBABE) interactions with −10 elements in the** ***ehxCABD*** **regulatory region in the presence of H-NS.** The panel shows an image of *ehxCABD* DNA cleavage products separated by electrophoresis on a denaturing acrylamide gel. DNA cleavage was mediated by 640 nM RNA polymerase associated with the σ^70^ RC461-FeBABE derivative that cleaves −10 hexamer sequences. Where present H-NS was pre-incubated with the DNA at concentrations of 235 nM, 470 M, 940 nM, 1645 nM or 2350 nM. The position of the *ehxCABD* promoter −10 hexamer is indicated. **B. Binding of H-NS to the** ***ehxCABD*** **F3 fragment.** The panel shows the result of a DNAse I footprint to monitor binding of H-NS to the *ehxCABD* DNA fragment. The gel is calibrated with a Maxim-Gilbert DNA sequencing reaction. H-NS was added at concentrations of 470 nM– 4700 nM. **C. Effect of H-NS on transcription start site selection at the** ***ehxCABD*** **regulatory region.** The panel shows the result of primer extension analysis using RNA extracted from strain M182 or the Δ*hns* derivative, carrying the *ehxCABD* F3 fragment cloned in pRW50, grown aerobically to mid-exponential phase (OD_650_ 0.4–0.6) in LB medium. The sizes of primer extension products were determined by calibration against size standards (A, C, G and T in Lanes 1–4). The brightness and contrast have been set differently for lanes 1–4 and 5–6 so that the primer extension products can be more easily compared to the marker lanes. The image otherwise represents a single continuous gel.

To more precisely understand the ternary H-NS-RNA polymerase-DNA complex we repeated our σ^70^RC461-FeBABE analysis. The data show that, in the presence of H-NS, the signal for RNA polymerase binding at the P*ehxCABD* −10 element is retained. Conversely, binding of RNA polymerase at adjacent sites is lost (Figure 4A). In a complementary experiment we used DNAse I footprinting to locate H-NS binding in the absence of RNA polymerase. The data show that H-NS recognises the same AT-rich region, extending from +10 to −30, as the transcriptional apparatus (Figure 4B). Thus, the binding sites for H-NS and RNA polymerase overlap. To assess how H-NS effects RNA polymerase interactions with P*exhCABD in vivo* we repeated our primer extension analysis. We used RNA extracted from wild type *E. coli* K-12 and cells lacking *hns*. As described above, RNA from wild type cells yielded two extension products of 155 and 154 nt in length (Figure 4C lane 5). These extension products were also observed when we analysed RNA from Δ*hns* cells (Figure 4C lane 6). Strikingly, RNA from Δ*hns* cells yielded a further 9 extension products of between 138 and 194 nt in length. These additional primer extension products align with the additional sites of RNA polymerase binding observed in Figure 4A. Finally, it is noteworthy that, in order to observe the primer extension products in Lane 6 of Figure 4C, we had to “overload” the sample onto the gel. This suggests that the net result of reduced RNA polymerase binding specificity is a reduction in transcription. Consistent with this, we observed reduced expression from the F3 fragment, in cells lacking H-NS, using our LacZ reporter assay (Figure S2).

### Transcription from P*ehxCABD* is inhibited by overlapping RNA polymerase binding sites

**Figure 5:**
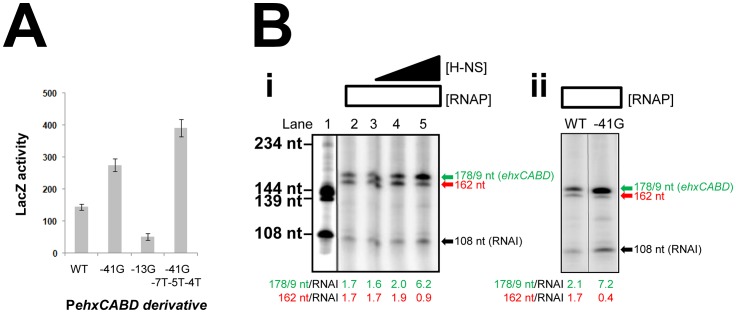
Transcription from P*ehxCABD* is inhibited by overlapping RNA polymerase binding sites. **A. Effects of mutations in P** ***ehxCABD*** **, and overlapping RNA polymerase binding sites.** The graph shows LacZ activity data for *E. coli* JCB387 cells carrying different F3::*lacZ* fusions in pRW50. **B. i) Stimulation of P** ***ehxCABD*** **by H-NS** ***in vitro*** **.** The figure shows the results of an *in vitro* transcription reaction calibrated with transcripts of known size from the *cbpA* regulatory region [35]. The 178 nt transcript initiates from P*ehxCABD* and the 108 nt RNAI transcript is an internal control. **ii) Stimulation of P** ***ehxCABD*** **by the -41G mutation** ***in vitro*** **.** The figure shows the results of an *in vitro* transcription assay comparing the wild type *ehxCABD* F3 fragment with a derivative carrying a mutation at promoter position -41.

Our data suggest that P*ehxCABD* is flanked by at least two overlapping elements that can bind RNA polymerase. If this model is correct there should be competition between RNA polymerase molecules for binding the various targets. A logical consequence of this competition would be reduced transcription from P*ehxCABD*. To test this model we disrupted either the P*ehxCABD* −10 hexamer or the overlapping RNA polymerase binding elements. The mutations utilised are illustrated in Figure 1D. Figure 5A shows LacZ activity data from wild type *E. coli* cells carrying the various promoter::*lacZ* fusions. The -41G mutation increases LacZ expression that is further increased by the -7T-5T-4T mutations. Conversely, the -13G mutation, in the canonical P*ehxCABD* −10 element, reduces LacZ expression.

We next sought to confirm the stimulatory effect of H-NS on specific recognition of P*ehxCABD* by RNA polymerase. Thus, we compared the effects of H-NS and the -41G mutation using *in vitro* transcription assays. The F3 DNA fragment was cloned upstream of the λ*oop* terminator in plasmid pSR. In the context of this construct P*ehxCABD* produces transcripts, of 178/179 nt in length, that can be quantified after electrophoresis. Additional transcripts, corresponding to the Δ*hns* primer extension products in Figure 4C, should also be generated. On this basis, we expected to detect an abundant 162 nt transcript (corresponding to the 138 nt extension product in Figure 4C) and scarce transcripts sized between 183 nt and 218 nt (equivalent to the primer extension products in the 159–194 nt range). The results of the analysis with and without H-NS are shown in Figure 5Bi alongside a set of “marker” transcripts (Lane 1). Lane 2 shows the result in the absence of H-NS. As expected we observed two intense bands corresponding to the 178/179 and 162 nt products. Note that because the bands in the 183–218 nt range are less abundant and poorly resolved in this assay they were not clearly visible. The 108 nt “RNAI” transcript is from the pSR replication origin and acts as an internal control. Addition of H-NS to the reactions specifically stimulated transcription from P*ehxCABD* (Lanes 2–5). Figure 5Bi shows the effect of the -41G mutation, it is indistinguishable from the effect of H-NS. Note that both the addition of H-NS, and the introduction of the -41G mutation, resulted in a decrease in the relative abundance of the 162 nt transcript compared to the RNAI control transcript (Figure 5B).

## Discussion

### Co-binding of H-NS and RNA polymerase at gene regulatory regions

**Figure 6:**
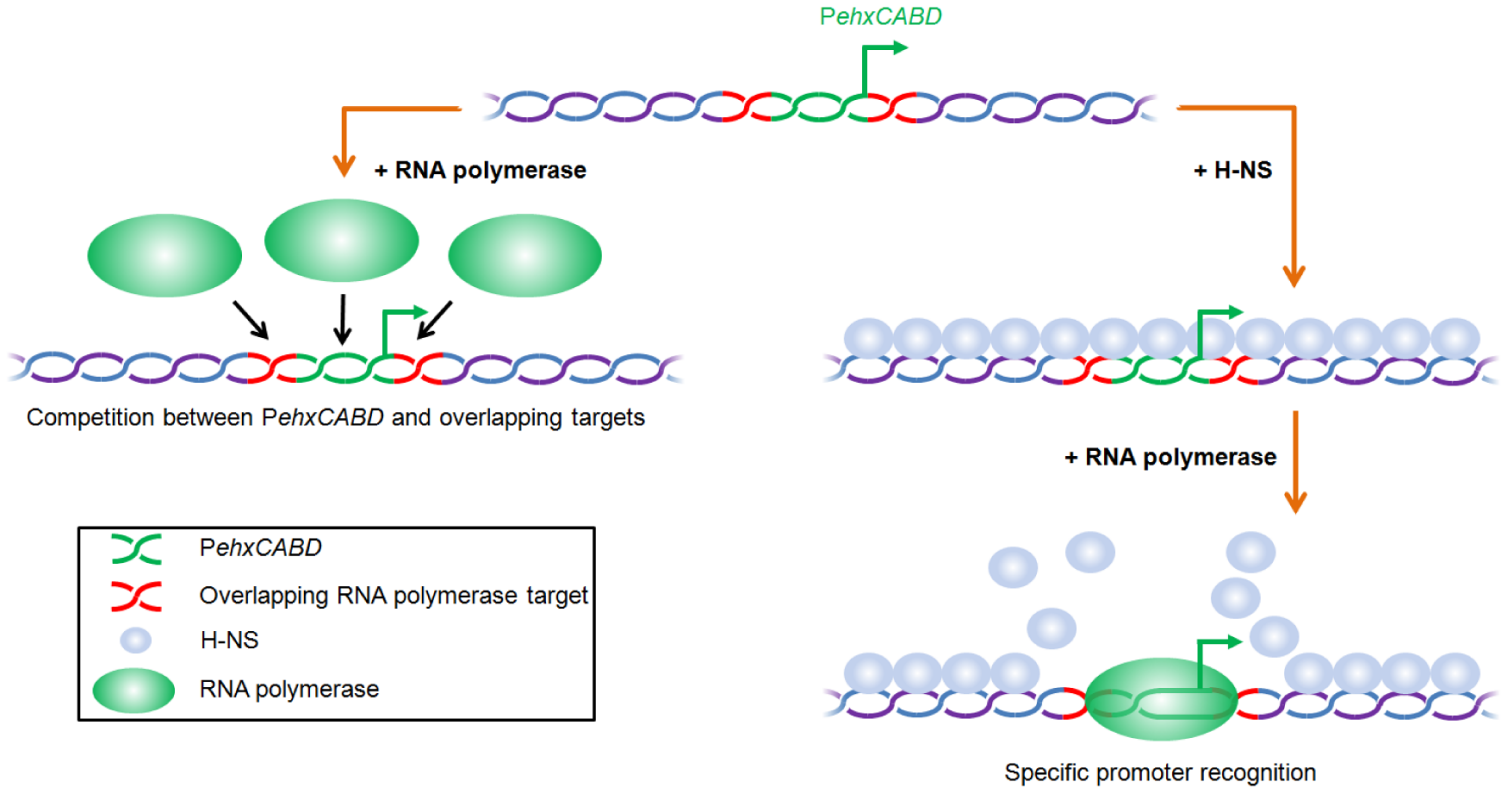
Model for H-NS induced specificity during interactions between RNA polymerase and AT-rich gene regulatory regions. In the absence of H-NS RNA polymerase competes with itself for binding to multiple overlapping targets (left hand side of figure). In the context of native nucleoprotein RNA polymerase must instead compete with H-NS. This results in preferential recognition of the canonical RNA polymerase binding target (right hand side of figure).

The data presented here demonstrate that nucleoprotein organisation, as well as primary DNA sequence, controls the specificity of regulatory DNA for RNA polymerase. In our model, RNA polymerase competes with itself for binding to AT-rich sequences overlapping P*ehxCABD* (Figure 6). In the context of native nucleoprotein this self-competition is negated. This is because RNA polymerase has instead to compete with H-NS (Figure 6). Hence, evolution of RNA polymerase binding targets likely involves a trade-off between attaining the optimal DNA sequence for correct chromosome folding and precise transcription initiation. We note the P*ehxCABD* has a consensus extended −10 element. Such sequences are incredibly rare, being found in only 3 of the 554 documented promoters in *E. coli* [23]. We speculate that, in very AT-rich gene regulatory regions, closer matches to the consensus RNA polymerase recognition elements are highly beneficial. Thus, in the presence of H-NS, RNA polymerase is able to recognise P*ehxCABD* because of its close similarity to a consensus promoter. Conversely, adjacent AT-rich sequences are ignored. Interestingly, the net effect of H-NS on transcription from P*ehxCABD* is positive and this results from correct positioning of RNA polymerase by H-NS (Figures 4 and 5). Park and co-workers [17] recently documented a mechanism for positive regulation of *malT* by H-NS. Although H-NS exerts its effect on *mal*T by binding the *malT* mRNA there are some clear parallels with the mechanism described here. Hence, the incoming ribosome is unable to correctly recognise the 5′ end of the *malT* mRNA because the Shine Dalgarno sequence is ambiguous. H-NS corrects mispositioning of the ribosome by binding to an adjacent AU-rich element. We note that the effect of H-NS on binding of RNA polymerase to P*ehxCABD* is similar to the effect of CRP on binding of RNA polymerase to the *acs*P2 promoter [24]. However, the molecular mechanisms underlying the effects are different. Hence, at *acsP2*, CRP makes direct contacts with RNA polymerase that ensure it engages the promoter precisely.

### Comparison with previous studies of the*ehxCABD* gene regulatory region

Rogers *et al.* [20] previously studied a 1338 bp DNA fragment carrying 126 bp of the *ehxCABD* gene regulatory region, the entire 516 bp *ehxC* gene, and 695 bp of *ehxA*. The fragment was fused to *lacZ* and, on detection of LacZ expression, it was concluded that a promoter must be located within the 126 bp regulatory section of the 1338 bp fragment. We show that, when examined in isolation, the 126 bp of DNA immediately upstream of *ehxC* is not able to promote transcription (see the F2 fragment in Figure 1). Similarly, no mRNA species were found to originate in this section of the regulatory region (highlighted blue in Figure S1). Thus, the only plausible explanation for the observations of Rogers *et al.* is that they unwittingly measured transcription from spurious promoters located within the AT-rich *ehxCABD* coding sequence. More recently, Iyoda and co-workers [21] examined the full *ehxCABD* regulatory region (similar to our F1 fragment). The authors found that deleting the upstream part of the regulatory region greatly reduced transcription. Building on the assumptions of Rogers *et al.* (2009) the authors presumed that they had removed the binding site for a transcriptional activator. A speculative binding site for the activator was identified; this sequence aligns with the P*ehxCABD* consensus extended −10 hexamer. Clearly, a more likely explanation is that Iyoda and co-workers had simply removed P*ehxCABD*. Taken together, these data suggest that control of *ehxCABD* expression is more complex than previously thought. In particular, the possibility that additional promoters exist within the *ehxCABD* coding sequence is intriguing [20]. Should any such promoters be repressed by H-NS, as suggested by Rogers *et al.* [20], this would further ensure specific transcription initiation from P*ehxCABD*. We also speculate that small differences in the DNA sequence of the *ehxCABD* regulatory region, in different *E. coli* isolates, may provide information about how H-NS regulated promoter regions evolve. Further biochemical and genetic dissection of the *ehxCABD* locus should provide the necessary insight.

## Methods

### Strains and plasmids

Wild type *E. coli* strains JCB387 and M182 have been described previously [25], [26]. The Δ*hns* M182 derivative (JRG4864) is described by Wyborn *et al.* [27]. Plasmids pRW50 and pLux are described by Lodge *et al.* [28] and Burton *et al.* [29] respectively. Plasmid pSR is described by Kolb *et al.* [30]. More detailed descriptions of strains and plasmids are provided in Table S1.

### Protein preparations and DNA footprinting

H-NS and RNA polymerase were prepared as described previously [22], [25]. DNA fragments for footprinting and EMSA experiments were derived from Qiagen maxi-preparations of plasmid pSR. Thus, the *ehxCABD* F3 fragment was excised from pSR by sequential digestion with *Hin*dIII and then *Aat*II. After digestion fragments were labelled at the *Hin*dIII end using [γ-^32^P]-ATP and polynucleotide kinase. DNAse I and KMnO_4_ footprints were then performed as described by Grainger *et al.* [25]. FeBABE footprinting reactions were completed according to the methodology of Bown *et al.* [22]. Radio-labelled DNA fragments were used at a final concentration of ∼10 nM. Note that, apart from the KMnO_4_ reactivity assays, all *in vitro* DNA binding reactions contained a vast excess (12.5 µg ml^−1^) of Herring sperm DNA as a non-specific competitor. We checked that our reaction conditions were meaningful by comparing the affinity of H-NS for P*ehxCABD* and the well-characterised H-NS target *proU*. We found that the affinity of H-NS for the two DNA fragments was similar in our conditions (Figure S3). Footprints were analysed on a 6% DNA sequencing gel (molecular dynamics). The results of all footprints and EMSA experiments were visualized using a Fuji phosphor screen and Bio-Rad Molecular Imager FX.

### *In vitro* transcription assays

The *in vitro* transcription experiments were performed as described previously Savery *et al.* [31] using the system of Kolb *et al.* [30]. A Qiagen maxiprep kit was used to purify supercoiled pSR plasmid carrying the different promoter inserts. This template (∼16 µg ml^−1^) was pre-incubated with purified H-NS in buffer containing 20 mM Tris pH 7.9, 5 mM MgCl_2_, 500 µM DTT, 50 mM KCl, 100 µg ml^−1^ BSA, 200 µM ATP, 200 µM GTP, 200 µM CTP, 10 µM UTP with 5 µCi [α-^32^P]-UTP. The reaction was started by adding purified *E. coli* RNA polymerase. Labelled RNA products were analysed on a denaturing polyacrylamide gel.

### Promoter DNA fragments and *in vivo* gene expression assays

Luciferase assays were done as described by Burton *et al.* [29] using *E. coli* O157:H7. β-galactosidase assays were completed using the protocols of Miller [32] with *E. coli* JCB387, M182 or the Δ*hns* derivative. All assay values are the average of three independent experiments and, in all cases, cells were grown aerobically, at 37°C, in LB media. The *ehxCABD* F1 fragment was synthesised by DNA2.0 (USA). The F3 fragment was generated using overlapping oligonucleotides (5′-ggctgcgaattctatcttacaaatcaatcatctgagtgttataatataacttagctgtgatatgtgtaagaatgtttaggcaat-3′ and 5′-cgcccgaagcttcatctctcccaaccaaaacaacattagcgataataatatattgcctaaacattcttacacatatca-3′). Similarly, F2 was generated using 5′-ggctgcgaattctgtttttagatgcttcttgcttaaaagaatataattcctgttcttttatatagagttctttaca-3′ and 5′-cgcccgaagcttcataatgtttaaacaaataagaaaattcagtaaatgtaaagaactctatataaaagaac-3′. Mutations were introduced using derivatives of these oligonucleotides. All *ehxCABD* regulatory region sequences are numbered with respect to the transcription start point (+1) and with upstream and downstream locations denoted by ‘−’ and ‘+’ prefixes respectively.

### Primer extension assays

Transcript start sites were mapped by primer extension, as described in Lloyd *et al.* [33], using RNA purified from strains carrying the F3 DNA fragment cloned in pRW50. The 5′ end-labelled primer D49724, which anneals downstream of the *Hin*dIII site in pRW50 was used in all experiments. Primer extension products were analysed on denaturing 6% polyacrylamide gels, calibrated with size standards, and visualized using a Fuji phosphor screen and Bio-Rad Molecular Imager FX.

### Chromatin immunoprecipitation

Chromatin Immunoprecipitation was done exactly as described previously [8], [34]. Briefly, formaldehyde crosslinked nucleoprotein, obtained from growing JCB387 cells carrying the F3 fragment in plasmid pRW50, was fragmented by sonication. Some of this sample was retained as the “total DNA” fraction. DNA cross-linked with RNA polymerase or H-NS was then precipitated using a rabbit polyclonal antibody against H-NS or an antibody against the RNA polymerase β-subunit (Neoclone). A control mock immunoprecipitation (with no antibody) was done in parallel. After immunoprecipitation the protein-DNA complexes were de-cross-linked and the DNA was recovered using a Qiagen PCR purification kit. Recovered DNA was resuspended in 50 µl of elution buffer and 1 µl of this solution was used as a template in a 50 µl PCR. The reactions were run for 28 cycles of amplification before 5 µl was loaded onto a 7.5% polyacrylamide gel. After electrophoresis PCR products were visualised with ethidium bromide. The oligonucleotide primers for amplification of the *yabN* [34] and *lacZ* [8] open reading frames, in their chromosomal context, have been described previously. To amplify P*ehxCABD* we used 5′-ggctgcctcgagtatcttacaaatcaatcatctgagtgttataatataacttagctgtga-3′ and 5′-cgcccgggatcccatctctcccaaccaaaacacattagcg-3′.

## Supporting Information

Figure S1 Location of the *ehxCABD* transcription start site in the context of the F1 fragment. The gel shows products from an mRNA primer extension analysis of the F1 fragment (Lane 5). The gel was calibrated using arbitrary size standards (A, C, G and T in Lanes 1–4). The expected location of the P*ehxCABD* transcription start site is highlighted in green. The transcription start site proposed by Iyoda *et al.* (2011) is highlighted in blue.
(PDF)

Figure S2 H-NS stimulates transcription from the F3 fragment. The graph shows LacZ activity data for *E. coli* M182 cells, and the Δ*hns* derivative, carrying the F3::*lacZ* fusion in pRW50.
(PDF)

Figure S3 Comparative affinity of H-NS for the *ehxCABD* F3 fragment and the *proU* locus. Results of an EMSA showing binding of H-NS (50 nM, 100 nM, 200 nM, 400 nM, 800 nM, 1000 nM and 2500 nM) to the *proU* locus and to the *ehxCABD* F3 fragment.
(PDF)

Table S1 Strains, plasmids and oligonucleotide sequences.
(DOCX)
