# Supplementary figures and images for: H-NS Can Facilitate Specific DNA-binding by RNA Polymerase in AT-rich Gene Regulatory Regions
Source: PLoS Genet. 2013 Jun 20;9(6):e1003589. doi: 10.1371/journal.pgen.1003589 (PMC3688479; doi:10.1371/journal.pgen.1003589)

Figure S1

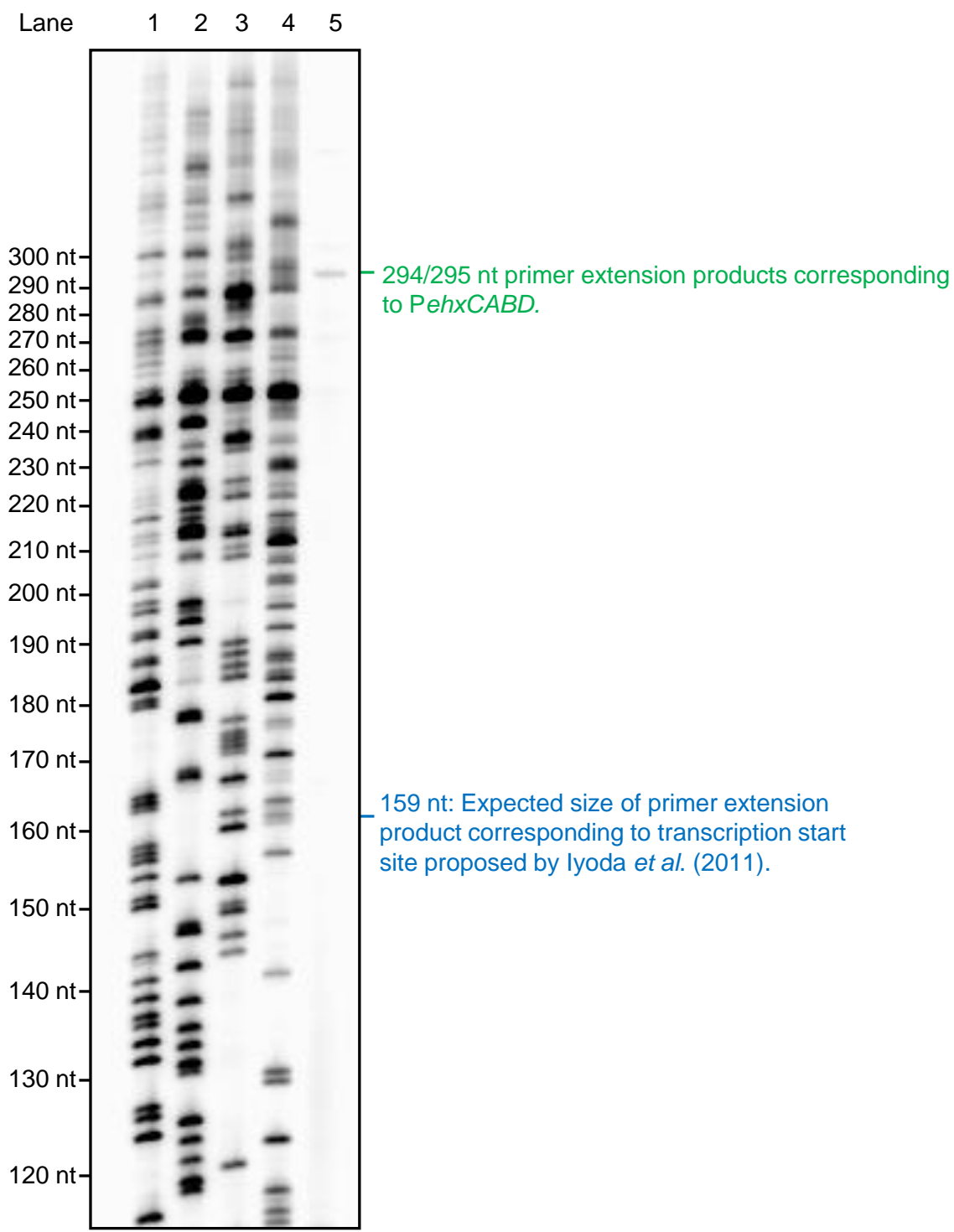

Supplement: Figure S1 — Location of the ehxCABD transcription start site in the context of the F1 fragment. The gel shows products from an mRNA primer extension analysis of the F1 fragment (Lane 5). The gel was calibrated using arbitrary size standards (A, C, G and T in Lanes 1–4). The expected location of the PehxCABD transcription start site is highlighted in green. The transcription start site proposed by Iyoda et al. (2011) is highlighted in blue. (PDF) [file pgen.1003589.s001.pdf]

**Figure S2**

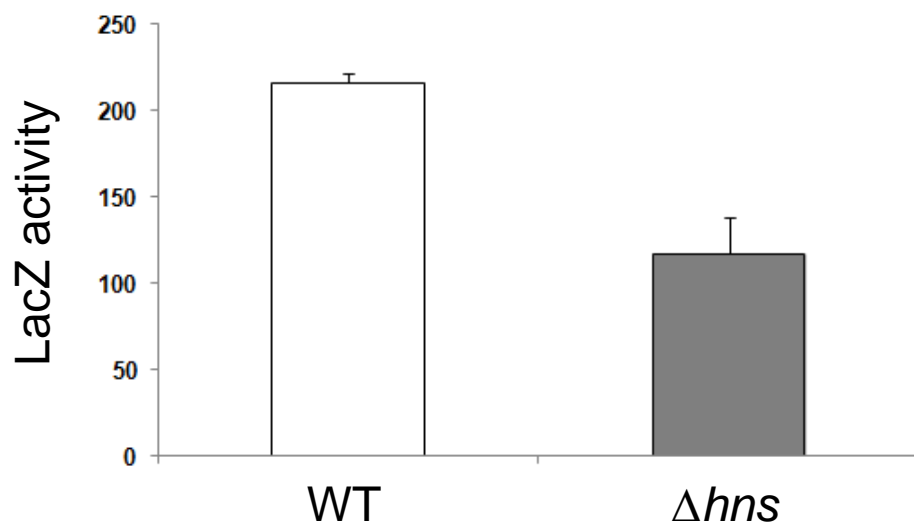

Supplement: Figure S2 — H-NS stimulates transcription from the F3 fragment. The graph shows LacZ activity data for E. coli M182 cells, and the Δhns derivative, carrying the F3::lacZ fusion in pRW50. (PDF) [file pgen.1003589.s002.pdf]

**Figure S3**

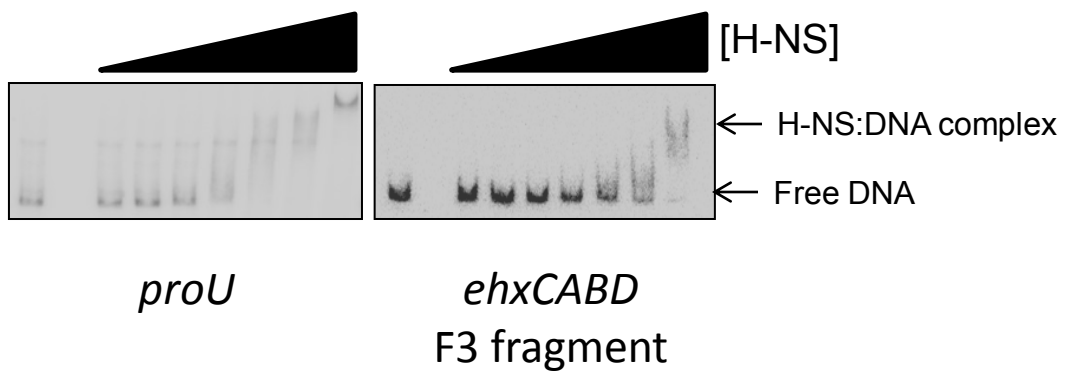

Supplement: Figure S3 — Comparative affinity of H-NS for the ehxCABD F3 fragment and the proU locus. Results of an EMSA showing binding of H-NS (50 nM, 100 nM, 200 nM, 400 nM, 800 nM, 1000 nM and 2500 nM) to the proU locus and to the ehxCABD F3 fragment. (PDF) [file pgen.1003589.s003.pdf]
