# Supplementary material for: H-NS Can Facilitate Specific DNA-binding by RNA Polymerase in AT-rich Gene Regulatory Regions
Source: PLoS Genet. 2013 Jun 20;9(6):e1003589. doi: 10.1371/journal.pgen.1003589 (PMC3688479; doi:10.1371/journal.pgen.1003589)
Supplement: Table S1 — Strains, plasmids and oligonucleotide sequences. (DOCX) [file pgen.1003589.s004.docx]

**Table S1: Strains and plasmids**

| **Name** | **Description** | **Source** |
| --- | --- | --- |
| **Bacterial strains** | | |
| JCB387 | Δ*nir* Δ*lac* | Page *et. al*. (1990) |
| M182 | Δ*lac* *galK* *galU* *strA* | Busby *et al*. (1983) |
|  |  |  |
| M182*Δhns* (JRG4864) | M182*Δhns* Cm^R^ | Wyborn *et al*. (2004) |
| **Bacterial plasmids** |  |  |
| pRW50 | Broad-host-range *lac* fusion vector for cloning promoters on *Eco*RI–*Hin*dIII fragments: contains the RK2 origin of replication and encodes TcR | Lodge *et. al*. (1992) |
| pSR | pBR322-derived plasmid containing an *Eco*RI–*Hin*dIII fragment upstream of the λ*oop* transcription terminator | Kolb *et. al*. (1995) |
| pLux | pCS26 derivative; STOP codons, ribosome binding site, and NcoI restriction site switch; parent plasmid for pLUX series | Burton *et al*. (2010) |

**REFERENCES**

Page L, Griffiths L, Cole JA. (1990) Different physiological roles of two independent pathways for nitrite reduction to ammonia by enteric bacteria. *Arch Microbiol*. **154:**349-54.

Lodge J, Fear J, Busby S, Gunasekaran P, Kamini NR. (1992) Broad host range plasmids carrying the *Escherichia coli* lactose and galactose operons. *FEMS Microbiol Lett.* **74:**271-6.

Kolb A, Kotlarz D, Kusano S, Ishihama A. (1995) Selectivity of the *Escherichia coli* RNA polymerase E sigma 38 for overlapping promoters and ability to support CRP activation. *Nucleic Acids Res*. **23:**819-26.

Wyborn NR, Stapleton MR, Norte VA, Roberts RE, Grafton J, and Green J (2004) Regulation of *Escherichia coli* hemolysin E expression by H-NS and Salmonella SlyA. J Bacteriol 186:1620–1628.

Busby S, Kotlarz D, and Buc, H (1983) Deletion mutagenesis of the *Escherichia coli* galactose operon promoter region. J. Mol. Biol. 167:259–274.

Burton NA, Johnson MD, Antczak P, Robinson A, Lund PA (2010) Novel aspects of the acid response network of E. coli K-12 are revealed by a study of transcriptional dynamics. J. Mol. Biol. 401:726-742.
